# Lessons learned from identifying clusters of severe acute respiratory infections with influenza sentinel surveillance, Bangladesh, 2009–2020

**DOI:** 10.1111/irv.13201

**Published:** 2023-09-22

**Authors:** Md Ariful Islam, Md Zakiul Hassan, Mohammad Abdul Aleem, Zubair Akhtar, Sukanta Chowdhury, Mustafizur Rahman, Mohammed Ziaur Rahman, Md Kaousar Ahmmed, Syeda Mah‐E‐Muneer, A. S. M. Alamgir, Shah Niaz Rubaid Anwar, Ahmed Nawsher Alam, Tahmina Shirin, Mahmudur Rahman, William W. Davis, Joshua A. Mott, Eduardo Azziz‐Baumgartner, Fahmida Chowdhury

**Affiliations:** ^1^ Infectious Diseases Division, icddr,b Dhaka Bangladesh; ^2^ Nuffield Department of Medicine University of Oxford Oxford UK; ^3^ School of Population Health University of New South Wales Sydney New South Wales Australia; ^4^ Biosecurity Program, Kirby Institute University of New South Wales Sydney New South Wales Australia; ^5^ Institute of Epidemiology, Disease Control and Research (IEDCR) Dhaka Bangladesh; ^6^ Global Health Development EMPHNET Dhaka Bangladesh; ^7^ Influenza Division Centers for Disease Control and Prevention (CDC) Atlanta Georgia USA

**Keywords:** Bangladesh, cluster, influenza, SARI, surveillance

## Abstract

**Background:**

We explored whether hospital‐based surveillance is useful in detecting severe acute respiratory infection (SARI) clusters and how often these events result in outbreak investigation and community mitigation.

**Methods:**

During May 2009–December 2020, physicians at 14 sentinel hospitals prospectively identified SARI clusters (i.e., ≥2 SARI cases who developed symptoms ≤10 days of each other and lived <30 min walk or <3 km from each other). Oropharyngeal and nasopharyngeal swabs were tested for influenza and other respiratory viruses by real‐time reverse transcriptase‐polymerase chain reaction (rRT‐PCR). We describe the demographic of persons within clusters, laboratory results, and outbreak investigations.

**Results:**

Field staff identified 464 clusters comprising 1427 SARI cases (range 0–13 clusters per month). Sixty percent of clusters had three, 23% had two, and 17% had ≥4 cases. Their median age was 2 years (inter‐quartile range [IQR] 0.4–25) and 63% were male. Laboratory results were available for the 464 clusters with a median of 9 days (IQR = 6–13 days) after cluster identification. Less than one in five clusters had cases that tested positive for the same virus: respiratory syncytial virus (RSV) in 58 (13%), influenza viruses in 24 (5%), human metapneumovirus (HMPV) in five (1%), human parainfluenza virus (HPIV) in three (0.6%), adenovirus in two (0.4%). While 102/464 (22%) had poultry exposure, none tested positive for influenza A (H5N1) or A (H7N9). None of the 464 clusters led to field deployments for outbreak response.

**Conclusions:**

For 11 years, none of the hundreds of identified clusters led to an emergency response. The value of this event‐based surveillance might be improved by seeking larger clusters, with stronger epidemiologic ties or decedents.

## 1 INTRODUCTION

While event‐based surveillance can be useful in identifying public health events of concern, it is unclear if hospital‐based sentinel surveillance can identify clusters of public health significance. Detection of epidemiologically linked clusters of severe acute respiratory infections (SARIs) may help public health officials respond to respiratory pathogens of pandemic potential.

The emergence of severe acute respiratory syndrome (SARS) led to the development revision of the International Health Regulations (IHR) 2005.^1^ IHR provides a legal framework for global health security and is a binding instrument for member states to develop core capacities to “Prevent, Detect and Respond” to future public health threats such as novel influenza viruses.^2^ One of the core capacities for IHR compliance is developing sensitive and timely surveillance systems that can detect and mitigate clusters of respiratory viruses with pandemic potential.^3^, ^4^, ^5^ Detection of unusual clusters of cases of respiratory disease, at the earliest stage of a potential outbreak, is important because it can allow public health officials to mobilize resources early to slow or stop the spread of disease before such viruses begin to circulate widely. Investigating clustered cases can be a useful event‐surveillance strategy because identifying and investigating individual suspected cases may not be an efficient use of resources, including laboratory resources unless cases are epidemiologically linked.^6^, ^7^, ^8^ While potentially useful, challenges may arise to efficiently identify novel viruses among clusters of respiratory viruses during seasonal epidemics.

Outbreak detection in sentinel surveillance is not a primary means of event‐based surveillance but may play a supportive role in meeting IHR core capacities by rapidly detecting new viruses that readily transmit between humans. For example, many influenza A (H7N9) cases in China that led to 616 deaths during 2013–2022^9^ were detected through a sentinel surveillance system for pneumonia of unknown etiology. Subsequent case investigations identified that some cases were part of clusters of human‐to‐human transmission,^10^ which modified health authorities' risk assessment of influenza A (H7N9). Similarly, nearly all clusters of human illness caused by influenza A (H5N1) virus have occurred among household members. While most people within these clusters had common source exposures, such as direct contact with sick or dead birds, cluster investigations have identified rare events of human‐to‐human transmission.^11^

In 2009, the government of Bangladesh strengthened several core capacities of IHR, including indicator and event‐based surveillance systems to detect respiratory clusters and respond rapidly to mitigate outbreaks. At the time, and in the wake of SARS, H5N1, and H1N1pdm09 outbreaks and pandemic, it was thought that cluster identification algorithms could improve the detection of outbreaks.^12^ The Institute of Epidemiology, Disease Control and Research (IEDCR) and International Centre for Diarrheal Disease Research, Bangladesh (icddr,b) embedded cluster identification and investigation functions within their national hospital‐based sentinel influenza surveillance.^13^

This evaluation describes the yield from 11 years of cluster event‐based surveillance embedded within the SARI indicator‐based sentinel surveillance system. Our goal was to determine the feasibility and usefulness of implementing the cluster algorithm in sentinel surveillance, as well as to explore signals that could efficiently differentiate clusters of novel viruses from seasonal viruses and conserve lab resources. We describe the epidemiological linkage between cases in clusters, the respiratory viruses identified, the timeliness of laboratory confirmation, and the action taken by health authorities upon identifying clusters.

## 2 METHODS

### 2.1 Hospital‐based influenza surveillance sites

In May 2007, IEDCR and icddr,b established the national hospital‐based influenza surveillance (HBIS) in 12 tertiary‐level hospitals across the country.^13^ HBIS aimed to: (1) monitor circulation and identify epidemiologic parameters of seasonal influenza in Bangladesh; (2) characterize the diversity of influenza strains; and (3) identify clusters of people with severe virus infections. During the May 2009–December 2020 period of evaluation, the number of hospitals in HBIS ranged from 7 to 14 (Table S1, Figure S1).

### 2.2 Case definition and study population

In each of the participating hospitals, surveillance physicians identified, consented, and enrolled all potential cases hospitalized with SARI based on WHO standardized case definitions.^14^, ^15^, ^16^, ^17^ Specifically, persons aged 5 or more years had SARI if they developed an acute respiratory illness with a history of fever or measured fever >38°C and cough or sore throat, requiring hospitalization, and presented within 7 days of symptoms onset for specimen collection.^13^, ^18^ Children aged less than 5 years with severe pneumonia (SP) (i.e., history of cough or difficulty breathing and at least one danger sign: chest indrawing, stridor in calm child, history of convulsions, inability to drink, lethargic, unconsciousness, or vomiting) had SARI if they required hospitalization and presented within 7 days of symptoms onset for specimen collection.^19^, ^20^ Starting in July 2016, the updated WHO case definition of SARI^16^ was used to enroll participants of all ages (i.e., an acute respiratory infection with a history of fever or measured fever ≥38°C and cough, with the onset of symptoms within the past 10 days of the date of specimen collection).^21^

### 2.3 Specimen and data collection from SARI and SP cases

After identifying SARI cases, surveillance physicians obtained their written informed consent to collect demographic (e.g., age, sex, and household information) and clinical information (e.g., symptoms, X‐ray findings, clinician's diagnosis, and history of lung disease) and oropharyngeal and nasopharyngeal swabs. Real‐time data were collected on handheld tablets using an Android‐based data collection software developed by icddr,b and uploaded to Microsoft SQL Server.

### 2.4 Cluster identification with epidemiological links

Cluster identification was performed by a full‐time field staff (one per surveillance site) who were hired to manage all aspects of the influenza surveillance. The additional workload for identifying clusters was determining if cases lived near each other and, if so, identifying an epidemiological link. This required scanning the SARI line list and discussions with patients. Each day, field staff recorded in a line list in which newly admitted SARI cases lived and the date of symptom onset. Surveillance physicians reviewed SARI line lists each day and administered short questionnaires that would enable the identification and characterization of the epidemiologic links between cases within clusters. From May 2009 to October 2010, SARI clusters were defined as ≥2 SARI cases who lived within <30 min walking distance (or within 3 km radius) and had illness onset within 7 days of each other. From November 2010 to December 2020, a cluster was redefined as ≥3 SARI cases who lived <30 min walk or 3 km from each other and had illness onset within 10 days of each other. Cases within a cluster were determined to be epidemiologically linked if they met at least one of the following conditions: (1) lived with another case in the same household or in neighboring households; (2) direct contact, indirect contact, or proximity to healthy, sick, or dead poultry; (3) a common exposure to a dead animal in their community; (4) any SARI deaths in the family or in the community within a 10‐day period; (5) international travel history or contact with international travelers within 2 weeks of symptom onset. Living in the same area defined a cluster but not an epidemiologic link. Surveillance physicians reported SARI clusters to IEDCR and icddr,b on the same day of identification and evaluated the cases each day during their hospitalization to monitor patients' recovery. In addition, they administered a cluster investigation form to collect standardized data about potential household, workplace, or community exposure to respiratory pathogens.

### 2.5 Laboratory analysis

Oropharyngeal and nasopharyngeal swabs were stored in nitrogen dry shippers on‐site at ≤−70°C, then transported to the icddr,b virology laboratory in Dhaka every 2 weeks. Scientists at the icddr,b laboratory tested the specimens for respiratory viruses by real‐time reverse transcriptase‐polymerase chain reaction (rRT‐PCR).^22^, ^23^, ^24^ All the specimens were tested for influenza A & B viruses, respiratory syncytial virus (RSV), human metapneumovirus (HMPV), adenovirus, and parainfluenza type 1, 2, and 3 viruses. Specimens that were positive for influenza A virus were further subtyped by rRT‐PCR to identify A (H1N1), A (H3N2), A (H5N1), or A (H1N1pdm09) subtypes.^25^, ^26^ If an influenza sample was found to be unsubtypable, an aliquot of the sample was sent to CDC laboratories in Atlanta for further characterization. In response to the emergence of coronavirus disease 2019 (COVID‐19), we used rRT‐PCR to detect severe acute respiratory syndrome coronavirus 2 (SARS‐CoV‐2) beginning in March 2020.

### 2.6 Deployment of assets for outbreak response

**FIGURE 1:**
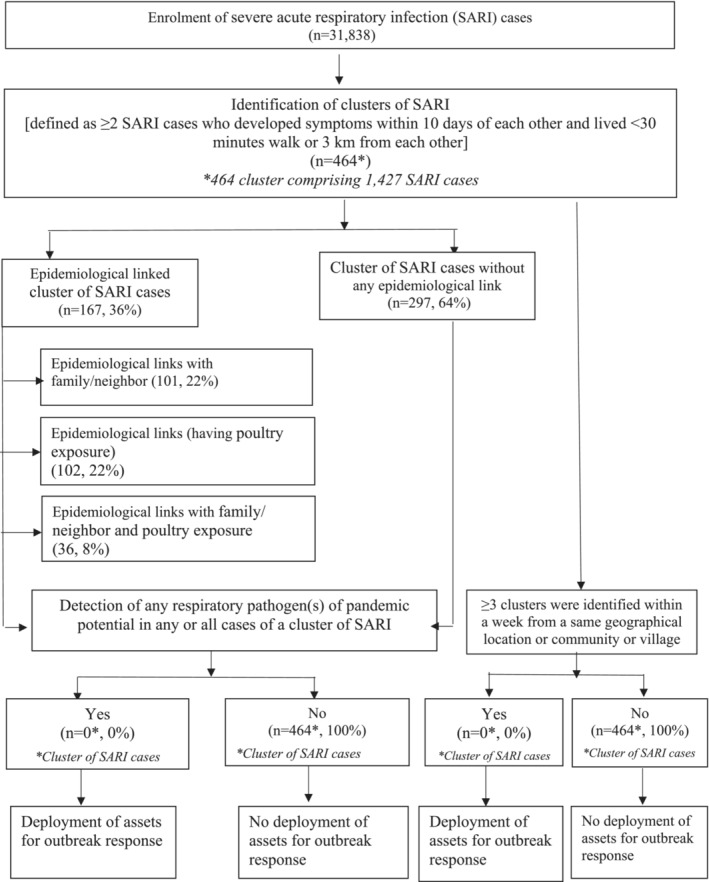
Flowchart of cluster of severe acute respiratory infection (SARI) cases identification and criteria of deployment of government assets for outbreak response.

Upon identifying a SARI cluster and after laboratory testing, local health authorities and IEDCR officials would assess the public health threat each seemed to represent and judge whether it merited the deployment of staff for a field outbreak investigation (Figure 1).

### 2.7 Data analysis

We described cases' demographic characteristics, clinical presentation, and rRT‐PCR test results using frequency and percentages and compared these using two sample z‐tests. To describe asymmetric distributed numerical variables including age in years, duration of hospitalization, time lag between the cases' symptom onset and the time of hospitalization, and number of household members, we used median and inter‐quartile range (IQR). The analyses were stratified by age groups because the incidence of respiratory viral infections among children <5 years of age and individuals aged ≥5 years is different, and most influenza A (H5N1) clusters have occurred exclusively among children aged <5 years. Data management and statistical analyses were conducted using Stata version 13, College Station, Texas 77845 USA.

### 2.8 Research ethics

The icddr,b ethics review committee approved the surveillance. The Human Research Protection Office at the US Centers for Disease Control and Prevention (CDC) reviewed and approved a continuing reliance on icddr,b ethics review.^27^, ^28^ All the patients or their caregivers provided written informed consent prior to specimen and data collection to participate in the surveillance.

## 3 RESULTS

### 3.1 Descriptive epidemiology of the clusters

**FIGURE 2:**
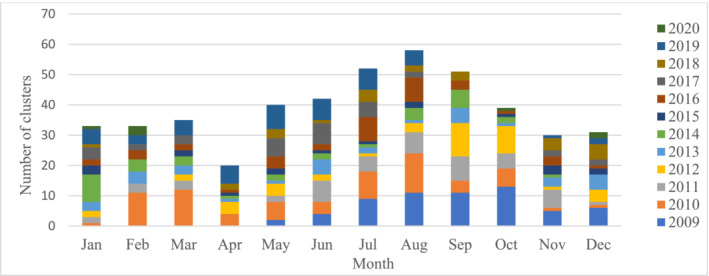
Monthly distribution of severe acute respiratory infection (SARI) clusters identified through hospital‐based influenza surveillance during May 2009–December 2020, Bangladesh.

From May 2009 to December 2020, we identified 31,838 SARI cases. Their median age was 20 years (IQR: 1.3–48 years) and 65% were male. Among the 31,838 SARI cases, surveillance physicians identified 464 clusters comprising 1427 cases; their median age was 2 years (IQR: 0.4–25), and 63% were male (Table S2). From May 2009 to October 2010, when SARI clusters were comprised of at least two SARI cases, 131 clusters were identified with 291 cases. When SARI clusters included at least three SARI cases between November 2010 and December 2020, 333 clusters were identified with 1136 cases. Among all the 464 clusters, 150 (32%) were comprised exclusively of young children aged <5 years, 101 (22%) comprised cases aged ≥5 years and 213 (46%) comprised cases aged <5 years and ≥5 years. Most clusters (276, 59%) had three cases, 109 (23%) clusters had two cases, and the rest (79, 17%) had four or more cases (Table S3). We identified an average of three clusters per month (range: 0–13) from all surveillance sites and 40 clusters per year (range: 7–72). The number of clusters per month was greatest from July to September (Figure 2). Most clusters (319, 69%) were within 5 km of the surveillance hospitals and 373 (80%) of the clusters were within 10 km of the surveillance hospitals.

### 3.2 Clinical symptoms, medical history, diagnosis, and illness outcome of the clustered cases

Aside from signs and symptoms in the SARI case definition, cases most commonly reported difficulty breathing during admission (1019, 71%). Children aged <5 years most commonly had chest indrawing during admission (659, 85%) (Table S2). Eighteen percent (135) of cases had at least one self‐reported preexisting condition such as asthma, chronic obstructive pulmonary disease (COPD), hypertension, diabetes, or ischemic heart disease (Table S2). Severe pneumonia (543, 70%) and pneumonia (61, 8%) were the two most common admission diagnoses among children aged <5 years. Viral fever (176, 27%), lower respiratory tract infection (166, 25%), and bronchial asthma (84, 13%) were the most common admission diagnoses among cases aged ≥5 years. Data about hospitalization outcomes at the time of discharge were available for 1419 (99.5%) of 1427 cases; 898 (63%) had fully recovered, 495 (35%) were still improving or discharged upon the request of the patient or the patient's guardian, 16 (1%) were transferred to another hospital, and 10 died (case‐fatality proportion 0.007) (characteristic of the cluster SARI cases who died during hospitalization mentioned in Table S4). Decedents belonged to 10 different clusters.

**FIGURE 3:**
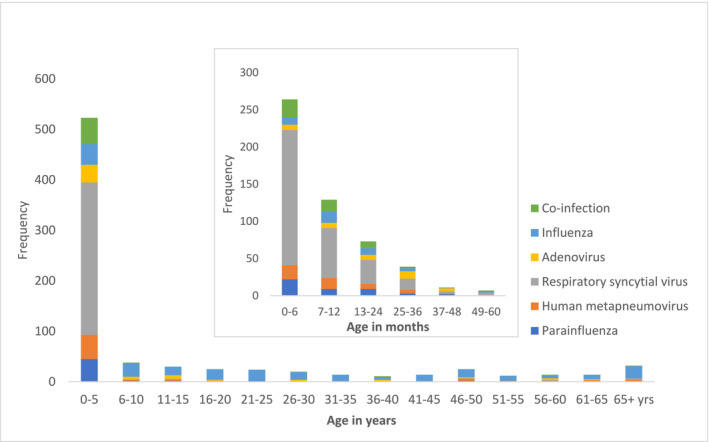
Age groups of cluster cases infected with respiratory viruses identified through hospital‐based influenza surveillance during May 2009–December 2020, Bangladesh.

Viral RNA was identified in samples from 795 (56%) of the 1427 cases. Of the viral RNA identified samples, influenza was detected in 238 (30%) cases (10% influenza A (H3N2), 10% (H1N1pdm09), 0% (H5N1), 0% (H7N9), and 10% influenza B). Other respiratory viruses were detected in 557 (70%) cases, and 59 (7%) had co‐detections (Table 1); SARS‐CoV‐2 was not detected in any cluster. Among the viral RNA identified cases, children aged <5 years most commonly had RSV (302/523, or 58% of infections), followed by HMPV (48/523, 9%), human parainfluenza virus (HPIV) (45/523, 9%), or adenoviruses (35/523, 7%). Of the viral RNA identified cases, persons aged ≥5 years most commonly had influenza (197/272, 72%) (Table 1, Figure 3).

**TABLE 1:** Cluster cases with laboratory‐confirmed respiratory viral pathogens and duration of hospitalization identified by hospital‐based influenza surveillance during May 2009–December 2020, Bangladesh.

	Proportion of respiratory virus positive cases			Duration of hospitalization in days, median
	SARI (aged <5 years) *N* = 523	SARI (aged ≥5 years) *N* = 272	SARI (all age groups) *N* = 795	
Respiratory virus	*n* (%)	*n* (%)	*n* (%)	*n* (IQR)
Influenza virus detected	41 (8)	197 (72)	238 (30)	3 (2–4)
Influenza A	30 (6)	128 (47)	158 (20)	3 (2–4)
Influenza B	11 (2)	69 (25)	80 (10)	3 (2–4)
Subtypes of influenza A				
Influenza A (H1N1pdm09)	12 (2)	67 (25)	79 (10)	3 (2–4)
Influenza A (H3N2)	18 (3)	61 (22)	79 (10)	3 (2–5)
Respiratory syncytial virus	302 (58)	10 (4)	312 (39)	4 (3–6)
Human metapneumovirus	48 (9)	16 (6)	64 (8)	4 (3–7)
Adenovirus	35 (7)	29 (11)	64 (8)	4 (2–5)
Human parainfluenza	45 (9)	13 (5)	58 (7)	5 (3–6)
Co‐infection	52 (10)	7 (3)	59 (7)	4 (3–6)
SARS‐CoV‐2	0 (0)	0 (0)	0 (0)	‐
No virus detected	249	383	632 (79)	4 (4–6)

Abbreviations: IQR, inter‐quartile range; SARI, severe acute respiratory infection; SARS‐CoV‐2, severe acute respiratory syndrome coronavirus 2.

In 290 (62%) clusters, cases in each cluster were positive for viruses. All cases in each cluster tested positive for RSV in 58 (13%) of 464 clusters, influenza in 24 (5%) clusters, HMPV in five (1%) clusters, HPIV in three (0.6%) clusters, and adenovirus in two (0.4%) clusters. In 82 (18%) clusters, cases were rRT‐PCR‐negative for tested viruses (Table S3). Among clusters where cases tested positive for the same pathogen, none had epidemiologic links (i.e., same family, travel history, or animal exposure) (Table 2).

**TABLE 2:** Clusters identified by proximity of cases' homes.

Clusters identified by proximity of cases' homes (*n* = 464)	Mode of epidemiological links	Lab results	
		Clusters in which all cases tested positive for any tested respiratory viruses	Clusters in which all cases tested positive for the same pathogen
Epidemiological linked clusters (*n* = 167, 36%)	Family/neighbor (101, 22%)	43 (9.27%)	0 (0%)
	Poultry exposure only (102, 22%)	38 (8.19%)	0 (0%)
	Family/neighbor + poultry exposure (36, 8%)	65 (14.01%)	0 (0%)
Clusters without epidemiological link (*n* = 297, 64%)		72 (24.2%)	98 (33.0%)

Most clusters (297, 64%) where cases came from within a 3 km radius were not epidemiologically linked. In 101 (22%) clusters, cases were part of the same family or next‐door neighbors. In 102 (22%) clusters, cases shared a common poultry exposure, and in 36 (8%) clusters, cases were part of the same household or next‐door neighbors and had poultry exposure (Table 2). Sixty‐nine (5%, 69/1427) cases reported poultry death in their community within 2 weeks of symptom onset (Table 3).

**TABLE 3:** Epidemiological factors of SARI cluster cases and linked with SARI clusters identified through hospital‐based influenza sentinel surveillance during May 2009–December 2020, Bangladesh.

Epidemiological factors	Number of cases *N* = 1427	Number of clusters *N* = 464
		*n* (%)
Close contact with family/neighbor **OR** had common poultry exposure within 2 weeks of symptom onset	768 (54)	167 (36)
Close contact with family/neighbor **AND** had common poultry exposure within 2 weeks of symptom onset	207 (15)	36 (8)
Close contact with family or neighbor within 2 weeks of symptom onset	400 (28)	101 (22)
A common poultry exposure within 2 weeks of symptom onset	575 (40)	102 (22)
Poultry death in their community within 2 weeks of symptom onset	69 (05)	0
Animal death in their community within 2 weeks of symptom onset	42 (03)	0
Family members died with SARI symptoms within 2 weeks of symptom onset	2 (0.1)	0
Any died with SARI symptoms in the community within 2 weeks of symptom onset	29 (02)	0
International travel within 2 weeks of symptom onset	3 (0.2)	0
Contact any person with international travel within 2 weeks of symptom onset	22 (1.5)	0

Abbreviation: SARI, severe acute respiratory infection.

None of the 464 clusters identified through the HBIS sentinel surveillance system led to the emergent deployment of government teams for outbreak investigation and response for multiple reasons: clusters were small, no single jurisdiction had ≥3 clusters identified within a week's time, and viruses detected had epidemic rather than pandemic potential (Figure 1). During 2012–2015, IEDCR investigated five respiratory illness clusters that were identified outside of the SARI surveillance system^29^; these clusters were detected by local authorities, health care providers, or the media and informally or formally reported to IEDCR.

### 3.3 Duration from symptom onset to hospitalization and laboratory confirmation

The median time between the cases' symptom onset and the time of hospitalization was 4 days (IQR: 3–5 days). The median duration of hospitalization among infected cases with any of the respiratory viruses was 4 days (IQR: 2–6 days). The median time between onset of the second and third cases that comprised the cluster was 4 days (IQR: 3–5 days). The median time between onset of symptoms in the index case and cluster detection was 4 days (IQR: 3–6 days). The median time between cluster identification and the availability of laboratory results was 9 days (IQR: 6–13 days).

## 4 DISCUSSION

While the event‐based surveillance algorithm embedded in the HBIS indicator‐based sentinel surveillance system identified hundreds of SARI clusters, after laboratory testing, none warranted further investigation. None of the clusters with epidemiologic links had cases that tested positive for the same respiratory virus. The algorithm did not identify influenza A (H5N1), A (H7N9), or emerging respiratory viruses. These pathogens, however, are exceedingly rare, and frequent detections are not expected; IEDCR responded to an average of one cluster of respiratory illnesses per year. Adjustments to the cluster case definition, such as deaths, could be useful to optimize the balance between the sensitivity and specificity of this event‐based surveillance algorithm.

The cluster identification algorithm was not useful in identifying cases of respiratory illness prioritized by health authorities for investigation. For example, less than one in three clusters were epidemiologically linked by family ties, and less than one in three had exposure to poultry. An evaluation of pneumonia surveillance in China also found low utility in using clusters to identify cases of SARS‐CoV and Middle East respiratory syndrome coronavirus (MERS‐CoV); no one in the clusters tested positive for either virus.^12^ Sentinel surveillance seems inefficient in detecting potential public health events of international concern and must be complemented by other systems as recommended by the WHO Mosaic Respiratory Surveillance Framework.^30^

While the current system was of limited use, modifications could be made to increase its value. Redefining the cluster definition to enhance specificity and prioritizing cluster specimens for laboratory testing could improve the system's focus on high‐risk scenarios in compliance with IHR. Strategies to improve the cost‐effectiveness of the algorithm might include seeking larger clusters, clusters with deaths, clusters of cases with stronger epidemiologic ties, or excluding clusters with children aged less than 2 years because of the high incidence of pediatric viral infections. Investigating fewer priority clusters might also reduce the time from sample collection to laboratory confirmation, which is crucial for pathogen confirmation, in better compliance with IHR timelines. If the algorithm can be altered to improve detection and the effort and cost of adding a cluster algorithm to the system was small (the workload for identifying clusters was minimal, and each site had a cluster on average of once per three months), then it may be worth implementing as an additional means to detect outbreaks. While our testing algorithm identified respiratory virus RNA in more than half of the clusters, laboratory confirmation was delayed according to IHR 2005 standards. IHR states signatories should assess the public health risk of the events within 48 h of their identification and report these to WHO within 24 h of their investigation.^1^ In our system, however, laboratory results were only available a median of 9 days after cluster identification or approximately 5 days later than what is recommended by IHR as a reasonable time to investigate, report, and mitigate an outbreak. The delay was likely caused by weekly or biweekly batch shipment of specimens to the central laboratory for testing. It is not surprising that low‐income countries have difficulties complying with IHR because of limited resources for sample logistics and testing.^31^, ^32^, ^33^ During response to COVID‐19, the Government of Bangladesh established laboratories that could perform rRT–PCR testing at regional sites for SARS‐CoV‐2; these laboratories could test SARI cluster samples to improve the surveillance system timeliness in better compliance with IHR.

While laboratory testing at district hospitals will likely improve timeliness, there are inherent delays in event identification through hospital‐based surveillance. Such systems identify cases once these have become sufficiently ill to seek hospital care. In HBIS, for example, the median time from illness onset to SARI hospitalization was already 4 days. Even if SARI clusters were identified within 24 h of admission and laboratory testing was available overnight, laboratory confirmation would occur approximately a week after illness onset. Furthermore, such events have to be sufficiently frequent in the community to be reliably identified by sentinel surveillance. It is therefore not surprising that during the 2009 pandemic, the first case of influenza A (H1N1pdm09) was first identified in Bangladesh among travelers through influenza‐like illness surveillance embedded within event‐based surveillance conducted by IEDCR, and detected 6 weeks later with HBIS.^34^, ^35^ IEDCR also detected the first cases of COVID‐19 through event‐based surveillance before these were detected in HBIS. Thus, while countries might choose to identify clusters of SARI as part of a broader event‐based surveillance strategy, they should not rely only on sentinel surveillance to detect respiratory viruses with pandemic potential.

It is noteworthy that 70% of the identified clusters occurred within 5 km of the surveillance hospitals and 80% occurred within 10 km of the surveillance hospitals. Such findings suggest that sites are adept at identifying cases and clusters that occur near hospitals. It is possible that sites are more likely to identify cases near the hospital because these are located near population centers, where most cases would occur, and that fewer cases would occur as population density wanes further away from population centers. It is also likely, however, that the systems are less adept at identifying cases that are further away because such cases are less likely to overcome the logistic barriers to seek care at the sentinel site. Cases that live more than 10 km from sentinel sites might choose care at a different hospital or be less likely to seek hospital care at all.^36^, ^37^ Such findings are consistent with previous surveillance evaluations of Bangladesh's hospital‐based surveillance^38^ and are important considerations when judging the representativeness and generalizability of national surveillance networks.

Many of the SARI clusters were exclusively among children aged <5 years who frequently tested positive for seasonal respiratory viruses that are common in this age group (e.g., RSV, HMPV, and HPIV). Such findings are consistent with previous investigations in Bangladesh and other low‐income countries.^39^, ^40^, ^41^

We did not identify viral RNA in approximately half of the SARI cluster cases. Such clusters could have been caused by common viruses that were not in our testing algorithm, including enteroviruses, seasonal coronaviruses, and bocaviruses, or non‐viral pathogens.

Our evaluation had several limitations. We sought to identify the surveillance system's ability to detect the exceedingly rare event of pathogen emergence. It is possible, however, that such events did not occur in Bangladesh during the evaluation period. In that scenario, even a perfect surveillance system would have been incapable of detecting an event that had yet to occur. Furthermore, we sought to quantify which proportion of clusters merited outbreak investigation but relied on IEDCR health authorities to judge what merited investigation; these criteria changed over time, and data were not available to determine the number of investigations. Next, our testing algorithms were limited by resource constraints such that we could only identify pathogens commonly tested through International Reagent Resource or commercially available kits. Access to pathogen discovery pipelines waxed and waned during the decade of surveillance making it difficult, if not impossible, to reliably identify emerging pathogens. Finally, the system was designed to detect clusters of SARI cases within the sentinel surveillance system, respiratory clusters with persons presenting without SARI or SARI clusters in areas of the country not covered by sentinel surveillance would necessarily have been missed.

## 5 CONCLUSION

While the cluster detection algorithm embedded in the sentinel surveillance system detected hundreds of clusters of respiratory illnesses, many were found to not harbor the same pathogen, and those that did, did not seem to merit outbreak investigations. Adjustments to the cluster case definition, for example, by seeking larger clusters with stronger epidemiologic ties or decedents, could be used to fine‐tune the signal‐to‐noise ratio and improve the system's relevance to emergency response. Furthermore, laboratory confirmation occurred 5 days after the IHR 2005‐recommended limit. Government of Bangladesh investments in subnational PCR testing during the COVID response might improve the timeliness of laboratory testing for epidemic‐prone viruses. Nevertheless, reviewing and revising pathogen discovery pipelines might be necessary to improve the likelihood of identifying pandemic threats early. Last, while countries like Bangladesh might choose to identify SARI clusters as part of a broader event‐based surveillance strategy, they should not rely only on such a strategy to detect respiratory viruses with pandemic potential.

## AUTHOR CONTRIBUTIONS

**Md Ariful Islam:** Conceptualization; data curation; formal analysis; investigation; methodology; project administration; resources; software; supervision; validation; visualization; writing—original draft; writing—review and editing. **Md Zakiul Hassan:** Conceptualization; formal analysis; methodology; writing—review and editing. **Mohammad Abdul Aleem:** Writing—review and editing. **Zubair Akhtar:** Writing—review and editing. **Sukanta Chowdhury:** Writing—review and editing. **Mustafizur Rahman:** Writing—review and editing. **Mohammed Ziaur Rahman:** Writing—review and editing. **Md Kaousar Ahmmed:** Data curation; resources; validation; writing—review and editing. **Syeda Mah‐E‐Muneer:** Writing—review and editing. **A.S.M. Alamgir:** Writing—review and editing. **Shah Niaz Md. Rubaid Anwar:** Writing—review and editing. **Ahmed Nawsher Alam:** Writing—review and editing. **Tahmina Shirin:** Writing—review and editing. **Mahmudur Rahman:** Writing—review and editing. **William W. Davis:** Conceptualization; methodology; writing—review and editing. **Joshua A Mott:** Writing—review and editing. **Eduardo Azziz‐Baumgartner:** Conceptualization; methodology; writing—original draft; writing—review and editing. **Fahmida Chowdury:** Conceptualization; funding acquisition; investigation; methodology; project administration; supervision; writing—original draft; writing—review and editing.

## CONFLICT OF INTEREST STATEMENT

None declared.

### PEER REVIEW

The peer review history for this article is available at https://www.webofscience.com/api/gateway/wos/peer-review/10.1111/irv.13201.

## DISCLAIMER

The opinions expressed by authors contributing to this journal do not necessarily reflect the opinions of the Centers for Disease Control and Prevention (CDC) or the institutions with which the authors are affiliated.

## Supporting information


**Figure S1:** Number of clusters within hospital catchment area.

**Table S1:** Identification of SARI clusters by hospital with the methodological changes of hospital‐based influenza surveillance in Bangladesh.

**Table S2:** Demographic and clinical data of clustered SARI cases identified through hospital‐based influenza surveillance in Bangladesh during May 2009–December 2020.

**Table S3:** Characteristics of SARI clusters identified through hospital‐based influenza surveillance in Bangladesh during May 2009–December 2020.

**Table S4:** Characteristic of the cluster SARI cases who died during hospitalization.

## Data Availability

Data generated during the study are subject to a data access policy of icddr,b and are available from icddr,b's research administration on reasonable request through the corresponding author.
