# Supplementary figures and images for: Lessons learned from identifying clusters of severe acute respiratory infections with influenza sentinel surveillance, Bangladesh, 2009–2020
Source: Influenza Other Respir Viruses. 2023 Sep 22;17(9):e13201. doi: 10.1111/irv.13201 (PMC10515138; doi:10.1111/irv.13201)

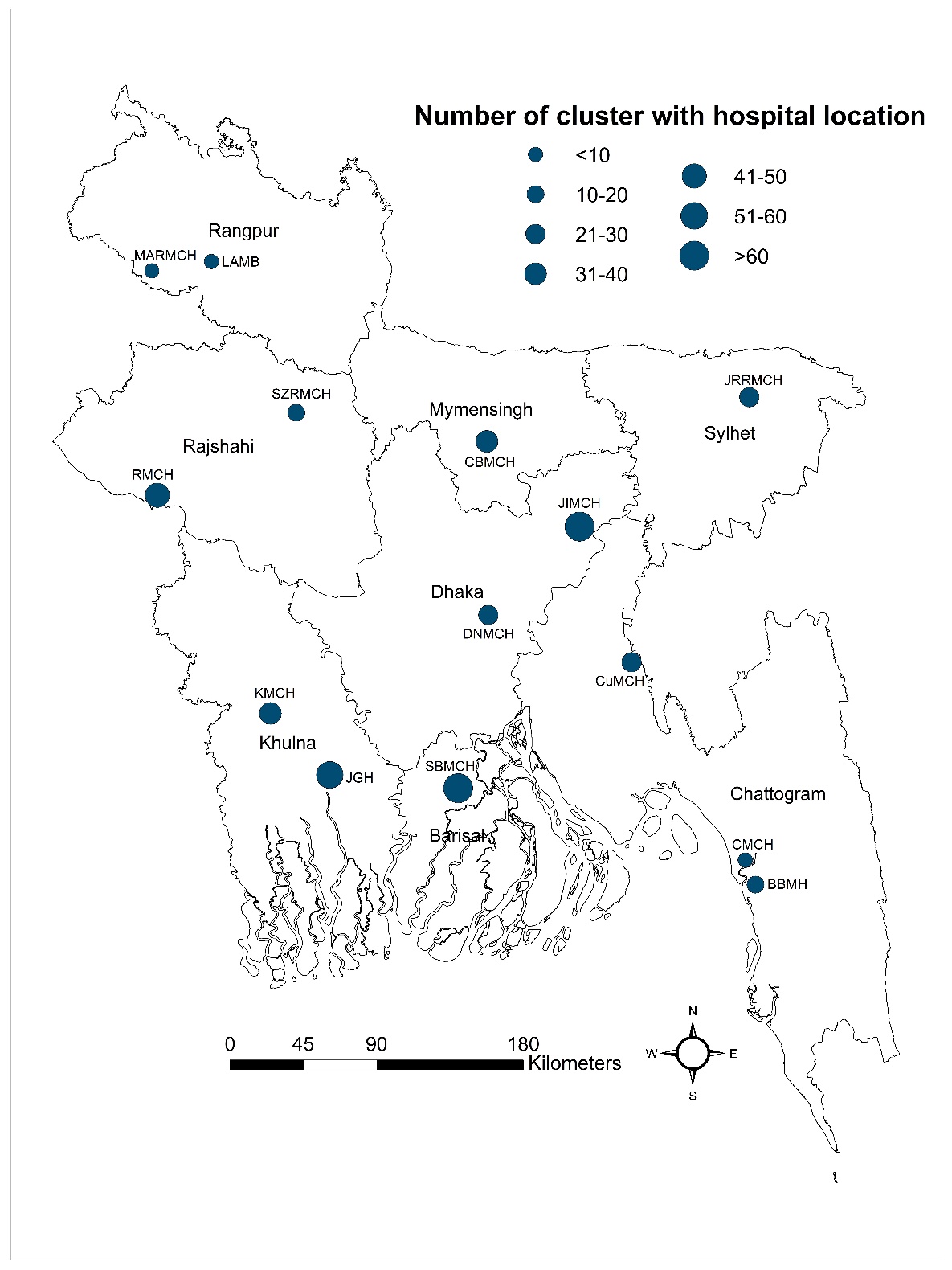


**Figure S1:** Number of clusters within hospital catchment area

Supplement: Supplementary file 1 — Figure S1: Number of clusters within hospital catchment area. [file IRV-17-e13201-s004.docx]
