# Supplementary material for: Lessons learned from identifying clusters of severe acute respiratory infections with influenza sentinel surveillance, Bangladesh, 2009–2020
Source: Influenza Other Respir Viruses. 2023 Sep 22;17(9):e13201. doi: 10.1111/irv.13201 (PMC10515138; doi:10.1111/irv.13201)
Supplement: Supplementary file 2 — Table S1: Identification of SARI clusters by hospital with the methodological changes of hospital‐based influenza surveillance in Bangladesh. [file IRV-17-e13201-s003.docx]

**Table S1: Identification of SARI clusters by hospital with the methodological changes of hospital-based influenza surveillance in Bangladesh**

| **Sl. #** | **Surveillance sites** (Hospitals) | **Location (District)** | **Years in operation** | **Participating wards of hospitals with years in operation** | | | | **SARI cases identified** | **SARI clusters identified** | **Cases in identified clusters** |
| --- | --- | --- | --- | --- | --- | --- | --- | --- | --- | --- |
|  |  |  |  | **Medicine and pediatric** | **ICU** | **CCU** | **Isolation wards*** |  |  |  |
| 1 | Dhaka National Medical College Hospital (DNMCH) | Dhaka | May 2009 – September 2017 | May 2009 – September 2017 | - | - |  | 1,474 | 29 | 116 |
| 2 | Community Based Medical College Hospital (CBMCH) | Mymensingh | May 2009 – September 2017 | May 2009 – September 2017 | - | - |  | 1,598 | 34 | 100 |
| 3 | Jahurul Islam Medical College Hospital (JIMCH) | Kishoregonj | May 2009 – December 2020 | May 2009 – December 2020 | January - December 2018 | January - December 2020 | June - December 2020 | 3,892 | 76 | 220 |
| 4 | Rajshahi Medical College Hospital (RMCH) | Rajshahi | May 2009 – December 2020 | May 2009 – December 2020 | January - December 2018 | January - December 2020 | June - December 2020 | 3,073 | 45 | 131 |
| 5 | Shaheed Ziaur Rahman Medical College Hospital (SZRMCH) | Bogura | May 2009 – September 2017 | May 2009 – September 2017 | January - December 2018 |  |  | 1,917 | 16 | 45 |
| 6 | LAMB Hospital (LAMB) | Dinajpur | April 2014 – September 2017 | April 2014 – September 2017 | - | - |  | 695 | 4 | 12 |
| 7 | Bangabandhu Memorial Hospital (BBMH) | Chattogram | April 2014 – September 2017 | April 2014 – September 2017 | - | - |  | 896 | 13 | 36 |
| 8 | Cumilla Medical College Hospital (CuMCH) | Cumilla | May 2009 – September 2017 & January 2018 – December 2020 | May 2009 – September 2017 & January 2018 – December 2020 | January - December 2018 | January - December 2020 | June - December 2020 | 2,795 | 25 | 67 |
| 9 | Khulna Medical College Hospital (KMCH) | Khulna | May 2009 – December 2020 | May 2009 – December 2020 | January - December 2018 | January - December 2020 | June - December 2020 | 2,651 | 53 | 148 |
| 10 | Jashore 250 bed General Hospital (JGH) | Jashore | May 2009 – September 2017 & December 2018 – December 2020 | May 2009 – September 2017 & December 2018 – December 2020 | January - December 2018 | January - December 2020 | June - December 2020 | 2,332 | 38 | 114 |
| 11 | Jalalabad Ragib-Rabeya Medical College Hospital (JRRMCH) | Sylhet | May 2009 – December 2020 | May 2009 – December 2020 | January - December 2018 | January - December 2020 | June - December 2020 | 2,411 | 26 | 81 |
| 12 | Sher-e-Bangla Medical College Hospital (SBMCH) | Barishal | May 2009 – December 2020 | May 2009 – December 2020 | January - December 2018 | January - December 2020 | June - December 2020 | 3,451 | 90 | 285 |
| 13 | Chattogram Medical College Hospital (CMCH) | Chattogram | April 2014 – December 2020 | April 2014 – December 2020 | January - December 2018 | January - December 2020 | June - December 2020 | 2,819 | 9 | 51 |
| 14 | M Abdur Rahim Medical College Hospital (MARMCH) | Dinajpur | April 2014 – December 2020 | April 2014 – December 2020 | January - December 2018 | January - December 2020 | June - December 2020 | 1,834 | 6 | 21 |
| Total: |  | | | | | |  | 31,838 | 464 | 1,427 |

*Specialised isolation wards established in March 2020 in response to the COVID-19 pandemic
