# Supplementary material for: Lessons learned from identifying clusters of severe acute respiratory infections with influenza sentinel surveillance, Bangladesh, 2009–2020
Source: Influenza Other Respir Viruses. 2023 Sep 22;17(9):e13201. doi: 10.1111/irv.13201 (PMC10515138; doi:10.1111/irv.13201)
Supplement: Supplementary file 3 — Table S2: Demographic and clinical data of clustered SARI cases identified through hospital‐based influenza surveillance in Bangladesh during May 2009–December 2020. [file IRV-17-e13201-s002.docx]

**Table S2:** Demographic and clinical data of clustered SARI cases identified through hospital-based influenza surveillance in Bangladesh during May 2009–December 2020

| **Characteristics** | **Cluster cases**  **N= 1,427** | **Influenza, N=238** | | **RSV,**  **N=312** | **HMPV,**  **N=64** | **Adenovirus, N=64** | **Parainfluenza 1,2 &3, N=58** | | **Co-infection***  **N=59** |  |
| --- | --- | --- | --- | --- | --- | --- | --- | --- | --- | --- |
|  | n (%) | n (%) | | n (%) | n (%) | n (%) | n (%) | n (%) | |  |
| **Demographics** | | |  |  |  |  |  |  |  |  |
| Age in years, median (IQR) | 2 (0.4-25) | 22 (8.0-45) | | 0.3 (0.2 – 0.8) | 0.8 (0.4-5.0) | 3.0 (1.3-17.5) | 0.9 (0.3-3.0) | | 0.6 (0.3-1.5) |  |
| Male | 906 (63) | 142 (60) | | 206 (66) | 42 (65) | 36 (56) | 39 (67) | | 48 (81) |  |
|  | | | | | | | | | |  |
| **Symptoms during presentation of all SARI cases** | | | | | | | | | |  |
|  | | | | | | | | | |  |
| Fever | 1,368 (96) | 238 (100) | | 282 (90) | 61 (94) | 61 (95) | 57 (98) | | 56 (95) |  |
| Cough | 1,414 (99) | 238 (100) | | 309 (99) | 64 (100) | 64 (100) | 58 (100) | | 59 (100) |  |
| Difficulty breathing, % | 1,019 (71) | 123 (52) | | 284 (91) | 56 (86) | 43 (67) | 47 (81) | | 53 (90) |  |
| Sore throat | 185 (13) | 59 (25) | | 4 (1) | 3 (5) | 10 (16) | 4 (7) | | 1 (2) |  |
|  |  |  | |  |  |  |  | |  |  |
| **Symptoms during presentation of SARI cases (aged < 5 years)** | | | | | | | | | | |
|  | **N=772** | **N=41** | | **N=302** | **N=48** | **N=35** | **N=45** | | **N=52** |  |
| Chest indrawing, % | 659 (85) | 29 (71) | | 273 (90) | 43 (90) | 27 (77) | 40 (90) | | 48 (92) |  |
| Unable to drink, % | 172 (22) | 13 (32) | | 58 (21) | 14 (29) | 4 (11) | 11 (24) | | 11 (21) |  |
| Vomiting, % | 130 (17) | 5 (12) | | 51 (17) | 10 (21) | 6 (17) | 11 (24) | | 10 (19) |  |
| Lethargy, % | 46 (6) | 5 (12) | | 10 (03) | 6 (13) | 3 (09) | 5 (11) | | 1 (02) |  |
| Stridor, % | 85 (11) | 5 (12) | | 22 (07) | 6 (13) | 6 (17) | 9 (20) | | 5 (10) |  |
|  |  |  | |  |  |  |  | |  |  |
| **Preexisting condition (Self-reported) ^†^ (n=769)** | | | | | | | | | |  |
| ≥ 1 preexisting condition | 135 (18) | 28 (4) | | 6 (1) | 4 (0.5) | 9 (1) | 2 (0.3) | | 2 (0.3) |  |
| Asthma | 69 (9) | 9 (1) | | 1 (0.1) | 3 (0.4) | 7 (1) | 2 (0.3) | | 1 (0.1) |  |
| COPD | 35 (5) | 8 (1) | | 4 (0.5) | 0 (0) | 1 (0.1) | 0 (0) | | 1 (0.1) |  |
| hypertension | 35 (5) | 11 (1) | | 1 (0.1) | 2 (0.3 | 2 (0.3) | 0 (0) | | 0 (0) |  |
| Diabetes | 18 (2) | 5 (1) | | 0 (0) | 2 (0.3) | 1 (0.1) | 0 (0) | | 0 (0) |  |
| ischemic heart diseases | 4 (1) | 2 (0.3) | | 1 (0.1) | 0 (0) | 0 (0) | 0 (0) | | 0 (0) |  |
| Other preexisting conditions | 8 (1) | 2 (0.3) | | 1 (0.1) | 0 (0) | 0 (0) | 0 (0) | | 0 (0) |  |
|  | **Chest x-ray findings, N=589 who had chest radiograph** | | | | | | | | |  |
|  | N=589 | N= 95 | | N= 127 | N=29 | N=23 | N=28 | | N=21 |  |
| Normal, n (%) | 282 (48) | 61(64) | | 49 (39) | 14 (48) | 7 (30) | 10 (36) | | 8 (38) |  |
| Lobar consolidation, n (%) | 172 (29) | 15 (16) | | 59 (47) | 9 (31) | 7 (30) | 9 (32) | | 11 (52) |  |
| Alveolar infiltrate, n (%) | 16 (3) | 3 (3) | | 1 (1) | 1 (3) | 1 (4) | 1 (3) | | 0 (0) |  |
|  |  |  | |  |  |  |  | |  |  |
| **Physician’s diagnosis, n (%)** | | | | | | | | | |  |
|  | | |  |  |  |  |  |  |  |  |
| Bronchiolitis | 43 (3) | 8 (3) | | 16 (5) | 2 (3) | 1 (2) | 3 (5) | | 2 (3) |  |
| Bronchial asthma | 93 (7) | 18 (8) | | 1 (0.3) | 3 (5) | 11 (17) | 2 (3) | | 1 (2) |  |
| Severe pneumonia | 562 (39) | 30 (13) | | 226 (72) | 40 (63) | 22 (34) | 24 (41) | | 43 (73) |  |
| Acute respiratory infection | 77 (5) | 8 (3) | | 19 (6) | 3 (5) | 4 (6) | 5 (9) | | 3 (5) |  |
| Respiratory tract infections | 170 (12) | 55 (23) | | 1 (0.3) | 7 (11) | 4 (6) | 2 (3) | | 2 (3) |  |
| Viral fever | 189 (13) | 56 (24) | | 7 (2) | 4 (6) | 10 (16) | 5 (9) | | 2 (3) |  |
| Chronic obstructive pulmonary disease | 61 (4) | 17 (7) | | 3 (1) | 0 | 1 (2) | 2 (3) | | 1 (2) |  |
| Encephalitis | 30 (2) | 0 (0) | | 16 (5) | 0 | 3 (5) | 1 (2) | | 1 (2) |  |
| Other diagnoses | 202 (14) | 46 (19) | | 23 (7) | 5 (8) | 8 (13) | 14 (24) | | 4 (7) |  |

*A case is co-infected with more than one virus

^†^Data collection on preexisting condition (self-reported) was initiated in May 2013 (n=769). Preexisting condition (chronic disease) includes Asthma, COPD, hypertension, Diabetes, ischemic heart diseases and other preexisting conditions
