# Supplementary material for: Lessons learned from identifying clusters of severe acute respiratory infections with influenza sentinel surveillance, Bangladesh, 2009–2020
Source: Influenza Other Respir Viruses. 2023 Sep 22;17(9):e13201. doi: 10.1111/irv.13201 (PMC10515138; doi:10.1111/irv.13201)
Supplement: Supplementary file 4 — Table S3: Characteristics of SARI clusters identified through hospital‐based influenza surveillance in Bangladesh during May 2009–December 2020. [file IRV-17-e13201-s001.docx]

**Table S3:** Characteristics of SARI clusters identified through hospital-based influenza surveillance in Bangladesh during May 2009–December 2020

| **Cluster composition** | **Number of clusters**  **N=464** | **Age in year, Median (IQR)** |
| --- | --- | --- |
|  | n (%) |  |
| SARI (all aged < 5 years) | 150 (32) | 0.5 (0.2-0.8) |
| SARI (all aged ≥ 5 years) | 101 (22) | 26 (18-50) |
| SARI (both<5 and ≥5 years) | 213 (46) | 4.5 (0.6-27) |
| **Number of case patients in each cluster** | | |
| SARI (All age groups) | | |
| 2 | 109 (23) | 0.8 (0.3-18) |
| 3 | 276 (59) | 2.5 (0.5-28) |
| ≥4 | 79 (17) | 1.7 (0.3 – 28) |
| **Viral etiology of cluster cases** | | |
| Respiratory syncytial virus | 58 (13) | 0.4 (0.2 - 0.8) |
| Influenza | 24 (5) | 22 (7-45) |
| Human metapneumovirus | 5 (1) | 0.8 (0.4-5) |
| Parainfluenza 1,2 & 3 | 3 (0.6) | 0.9 (0.1-3) |
| Adenovirus | 2 (0.4) | 3 (1.3-18) |
| SARS-CoV-2 | 0 (0) | - |
| None of the tested pathogens identified | 82 (18) | - |
| Partially infected*** | 290 (62) | - |

**A cluster is partially infected when all of the cases within the cluster were not infected with the same virus or cases were infected with different viruses*
