# Supplementary material for: Lessons learned from identifying clusters of severe acute respiratory infections with influenza sentinel surveillance, Bangladesh, 2009–2020
Source: Influenza Other Respir Viruses. 2023 Sep 22;17(9):e13201. doi: 10.1111/irv.13201 (PMC10515138; doi:10.1111/irv.13201)
Supplement: Supplementary file 5 — Table S4: Characteristic of the cluster SARI cases who died during hospitalization. [file IRV-17-e13201-s005.docx]

**Table S4:** Characteristic of the cluster SARI cases who died during hospitalization

| **Characteristics** | **Number of death cases** |
| --- | --- |
|  | **N=10** |
|  | n (%) |
| Median age (IQR), years | 3.7 (0.18-55.0) |
| Sex |  |
| Male | 7 (70) |
| Diagnosis |  |
| Pneumonia | 5 (50) |
| Bronchial asthma | 1 (10) |
| Chronic obstructive pulmonary disease | 1 (10) |
| Viral fever | 1 (10) |
| Other diagnoses | 2 (20) |
| Virus detected |  |
| Influenza A/H1N1pdm09 | 1 (10) |
| Human parainfluenza viruses | 1 (10) |
| Respiratory syncytial virus | 1 (10) |
| Adenovirus | 1 (10) |
| Co-infected with both influenza A/H1N1pdm09 and adenovirus | 1 (10) |
| None | 5 (50) |
